# Trends in nontraumatic intestinal perforation-related mortality among adults in the United States from 1999 to 2020: A nationwide CDC WONDER analysis

**DOI:** 10.1097/MD.0000000000048931

**Published:** 2026-05-22

**Authors:** Onyekachi Emmanuel Anyagwa, Hashim Mohamed Siraj, Hanzala Zahid, Oluwatoyin Adalia Dairo, Muhammad Husnain Ahmad, Muskan Joshi, Mohammad Aamir Qayyum Sarguroh Khan, Anas Abdulkader, Masab Ali, Abhirami Babu, Nivedita Pant, Areeba Zubair, Chinedu Elvis Nwaduru

**Affiliations:** a Ivane Javakhishvili Tbilisi State University, Tbilisi, Georgia; b Punjab Medical College, Faisalabad, Punjab, Pakistan; c School of Medicine, New Vision University, Tbilisi, Georgia; d Department of Medicine, Tentishev Satkynbai Memorial Asian Medical Institute, Gagarina, Kant, Kyrgyzstan; e Tbilisi State Medical Institute: Tbilisi State Medical University, Tbilisi, Georgia; f Al Maaref University, Beirut, Lebanon; g Department of Internal Medicine, Punjab Medical College/Faisalabad Medical University, Faisalabad, Punjab, Pakistan; h College of Medicine, Our Lady of Fatima University, Valenzuela, Philippines; i David Tvildiani Medical University, Tbilisi, Georgia; j University of Utah Health, Salt Lake City, UT.

**Keywords:** CDC WONDER, epidemiology, gastrointestinal emergency, mortality trends, nontraumatic intestinal perforation, public health surveillance, United States

## Abstract

To characterize mortality trends associated with nontraumatic intestinal perforation (NIP) in the United States, which continue to remain poorly defined. NIP is defined as the loss of continuity of the bowel wall in which external trauma as an etiology has been excluded. NIP is a serious gastrointestinal emergency with limited national mortality trend data, particularly across various demographic and regional subgroups. NIP-related mortality data were extracted from the Centers for Disease Control and Prevention Wide-ranging Online Data for Epidemiologic Research database from 1999 to 2020. We analyzed age-adjusted mortality rates (AAMR) per 100,000 individuals and annual percent change (APCs) with 95% confidence interval (CI) in patients ≥ 15 years based on gender, race and geographical region. Overall, there were 122,161 NIP-related deaths among patients aged ≥ 15 years, with an increase in AAMR from 1.90 in 1999 to 2.0 in 2009 (APC: 0.19; CI: −0.33 to 0.71), and a gradual increase to 2.40 in 2020 (APC: 1.26; CI: 0.80 to 1.71). AAMR was lower in females compared to males in 1999 (2.0 vs 1.90; CI: 1.90 to 2.0). However, in 2020, AAMR values increased in females (2.20 vs 2.0; CI: 2.40 to 2.60; *P* < .05). AAMRs were higher in patients reported as non-Hispanic compared to Hispanic/Latinos (2.10 vs 1.50; CI: 2.10 to 2.10; *P* < .05). Variation in AAMR values across census regions was not significant. NIP-related AAMR was higher in nonmetropolitan than metropolitan areas (2.90 vs 1.90; CI: 2.10 to 2.10; *P* < .05). NIP-related mortality has risen among patients’ ≥15 years. Higher AAMRs in females, non-Hispanic individuals, and nonmetropolitan populations highlight the need for targeted preventive efforts.

## Graphical abstract

**Figure:**
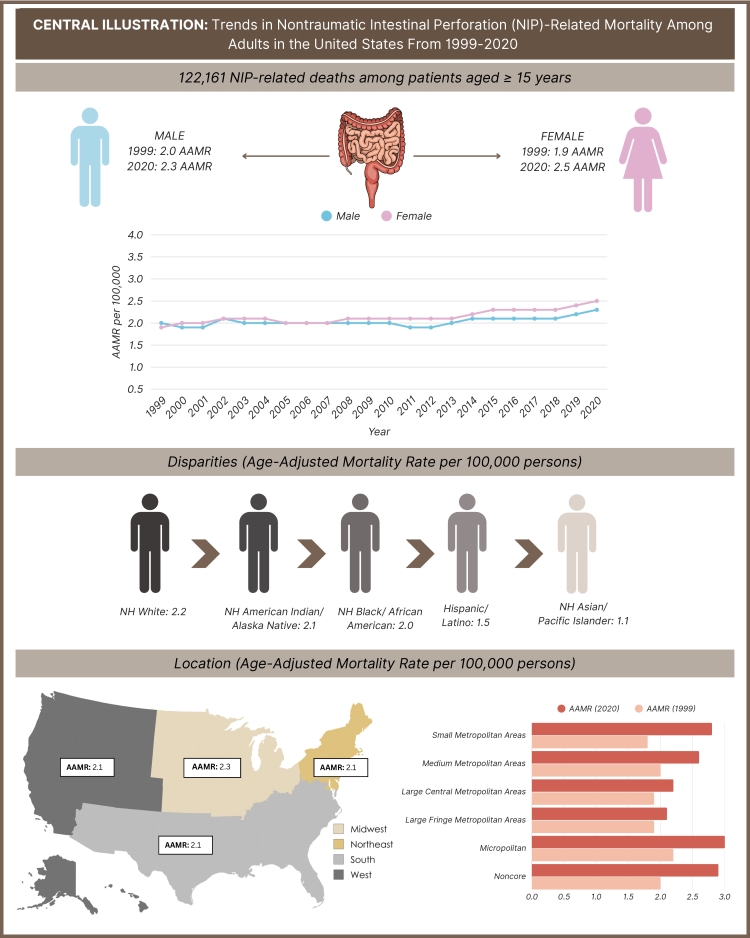

**Highlights**

● Over 122,000 deaths from nontraumatic intestinal perforation (NIP) occurred among adults aged ≥ 15 years between 1999 and 2020 in the United States.
● The age-adjusted mortality rate (AAMR) continued to remain stable from 1999 to 2009 (annual percent change [APC]: 0.19; 95% CI: −0.33 to 0.71), but increased significantly after 2009, reaching 2.40 per 100,000 by 2020 (APC: 1.26; 95% CI: 0.80–1.71).
● Males had a higher AAMR than females; with AAMR rising from ~4.0 to 5.0 per 100,000, while females remained around 2.0 with a gradual increase post-2014.
● Hispanic or Latino populations had the highest AAMRs, peaking at 11 per 100,000, followed by non-Hispanic White and Black populations; Asian/Pacific Islanders and American Indians had the lowest rates.
● AAMR varied with urbanization, with micropolitan areas experiencing the highest rise, particularly after 2015; large central metro areas had the lowest and most stable rates (APC: −0.43).
● Geographical variation was significant, with West Virginia and Iowa showing the highest AAMRs (~4 per 100,000), while Mississippi, Florida, and Kansas had the lowest (<1.5 per 100,000), indicating regional health disparities.
● A sharp surge in AAMRs across all demographics was observed in 2020, suggesting an external influence, potentially the COVID-19 pandemic.

## 1. Introduction

Nontraumatic intestinal perforation (NIP) is a rare but devastating gastrointestinal emergency, associated with significant morbidity and high mortality, especially in the absence of prompt diagnosis and surgical intervention.^[1,2]^ Unlike traumatic perforations resulting from blunt or penetrating abdominal injuries, NIP arises from intrinsic pathological processes that weaken the integrity of the bowel wall, eventually leading to full-thickness rupture. The etiology of NIP varies considerably across geographical regions and income levels, influenced by infectious burden, dietary habits, access to healthcare, and procedural exposure. In low- and middle-income countries, NIP is often the result of infectious conditions such as *Mycobacterium tuberculosis*, *Salmonella typhi*, or parasitic infestations.^[1,3]^ In contrast, in high-income nations such as the United States (US), NIP is more commonly attributed to noninfectious causes including inflammatory bowel disease, ischemic enterocolitis, diverticulitis, malignancy, and iatrogenic complications during colonoscopic or surgical procedures.^[3,4]^

Although NIP remains a relatively rare diagnosis, its incidence is far from trivial due to the disproportionately high case fatality rate. The small intestine is most frequently involved, with clinical presentation typically delayed and nonspecific, resulting in diagnostic uncertainty and delayed intervention.^[2,5]^ Left untreated, perforation may lead to generalized peritonitis, intra-abdominal sepsis, abscess formation, systemic inflammatory response syndrome, and multi-organ failure, pathways that rapidly culminate in death if not aggressively managed.^[6]^ Emergent surgical intervention remains the cornerstone of therapy. Depending on the patient’s clinical status and intraoperative findings, procedures may include primary closure of the defect, segmental bowel resection with anastomosis, or temporary fecal diversion with creation of an ostomy.^[5,7]^ More recently, minimally invasive techniques such as laparoscopic repair, along with principles of damage control surgery and early goal-directed sepsis management, have shown promise in improving short-term postoperative outcomes, particularly in stable patients.^[6,7]^

Despite improvements in perioperative care, outcomes for NIP remain poor, especially among older adults and those with multiple comorbidities. Several institutional and retrospective studies have explored short-term outcomes and predictors of mortality, yet these findings are often limited by small sample sizes and lack of generalizability.^[4,8,9]^ Moreover, disparities in outcomes based on sociodemographic characteristics, including sex, race, geographic region, and level of urbanization, remain poorly understood. Emerging literature has highlighted the role of systemic inequities in healthcare access, late presentation to care, and variations in hospital resources and infrastructure as contributing factors to these disparities.^[10–12]^ For instance, patients residing in rural or micropolitan areas may face delays in diagnosis due to limited access to imaging, a shortage of surgical specialists, and longer transport times to tertiary care centers.^[13,14]^ Lifestyle factors such as tobacco use and alcohol abuse, which differ in prevalence across populations, further compound risk for complications such as peptic ulcer perforation, bowel ischemia, and poor surgical tolerance.^[10,11]^ This study aims to uncover demographic and regional disparities in NIP-related mortality among adults in the U.S. from 1999 to 2020, using the CDC WONDER (Centers for Disease Control and Prevention Wide-Ranging Online Data for Epidemiologic Research) database, to guide clinicians, health systems, and policymakers in developing data-driven strategies to reduce disparities and improve NIP-related outcomes.^[4]^

## 2. Methods

### 2.1. Study setting and population

In this descriptive study, death certificate data were retrieved from the CDC WONDER database and examined from 1999 to 2020 for mortality related NIP.^[4]^ Death certificates where NIP was listed as either the underlying cause or contributing cause of death were included, and identified using the International Statistical Classification of Diseases and Related Health Problems-10th Revision code K63.1 (perforation of intestine [nontraumatic]). Prior studies using the CDC WONDER database have validated these codes to accurately capture NIP-related mortalities.^[5]^ This dataset includes cause-of-death information from death certificates for all the US, compiled through the National Vital Statistics System and the Vital Statistics Cooperative Program. The multiple Cause-of-Death Public Use record death certificates were studied to select NIP related deaths, which were identified as either a contributing or an underlying cause of death. The study population consisted of individuals aged 15 years and older. This study follows the Strengthening the Reporting of Observational Studies in Epidemiology guidelines in reporting.^[6]^

### 2.2. Data abstraction

**Figure 1.**
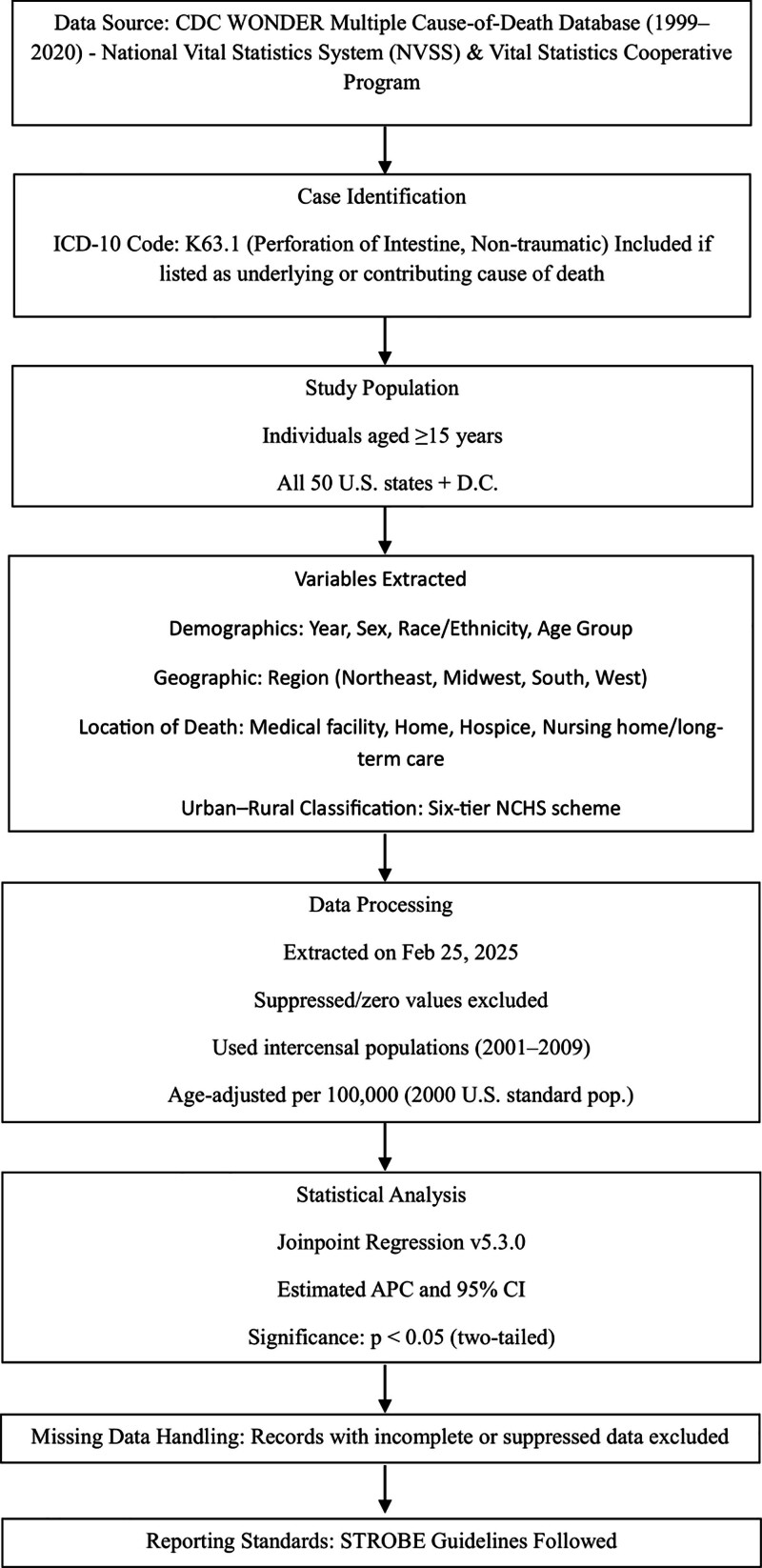
Schematic summary of the study. APC = annual percent change, CDC WONDER = Centers for Disease Control and Prevention Wide-ranging Online Data for Epidemiologic Research, ICD-10 = International Statistical Classification of Diseases and Related Health Problems, 10th Revision, STROBE = Strengthening the Reporting of Observational Studies in Epidemiology.

Data for population size, year, location of death, demographics, and urban-rural classification were abstracted from the CDC WONDER database on February 25, 2025, at 6:00 pm. Demographics included year, sex, race/ethnicity, and location of death. Age groups included were 15 to 24 years, 25 to 34 years, 35 to 44 years, 45 to 54 years, 55 to 64 years, 65 to 74 years, 75 to 84 years, and 85+ years. Race/ethnicity was classified as non-Hispanic (NH) White, NH Black or African American, Hispanic or Latino, NH American Indian or Alaskan Native, and NH Asian or Pacific Islander. Location of death included medical facilities (outpatient, emergency room, inpatient, death on arrival, or status unknown), home, hospice, and nursing home/long-term care facility. This information relies on reported data on death certificates and has been used in previous analyses of the WONDER database. The National Center for Health Statistics Urban-Rural Classification Scheme groups U.S. counties into 6 levels based on population size and metropolitan status, namely, Large Central Metro (counties within metropolitan areas with populations of 1 million or more), Large Fringe Metro (counties in the suburban periphery of large metro areas, also with populations of 1 million or more), Medium Metro (250,000–999,999), Small Metro (50,000–249,999), Micropolitan (10,000–49,999), and NonCore (fewer than 10,000), as defined by the 2013 U.S. Census classification.^[7]^ Regions were classified into Northeast, Midwest, South, and West according to the U.S Census Bureau guidelines.^[8]^ Specific parameters, including the 2000 U.S. standard population reference, per 100,000 rate calculations, grouping by year, and data display preferences by inclusion of totals, exclusion of zero values, and suppressed data, were applied to ensure consistency.^[9]^ The default intercensal populations for 2001 to 2009 (excluding infant age groups) were used. All retrieved data were systematically compiled and exported into a tabulated master file (Excel spreadsheet) for further statistical interpretation (Fig. 1).

## 3. Statistical analysis

To examine national trends in mortality related to NIP, we calculated age-adjusted mortality rates (AAMRs) per 100,000 individuals from 1999 to 2020, standardizing to the year 2000 US population as the baseline, to account for differences in age distribution over time. To quantify national annual trends, the Joinpoint regression program (version 5.3.0) was used to assess trends in AAMRs and estimate annual percent change (APC) with 95% confidence intervals (CIs).^[10]^ This method identifies significant changes in AAMR over time by fitting log-linear regression models where temporal variation occurred. APCs were classified as increasing or decreasing if the slope representing the change in mortality was found to be significantly different from zero using 2-tailed *t*-testing. A zero slope indicated no change in mortality over time, while a positive slope signified an increase in annual NIP-related mortality trends. A *P*-value of < .05 was considered statistically significant.

## 4. Results

In total, there were 122,161 NIP-related deaths among patients aged 15 years and older over the study period. The AAMR increased from 1.90 per 100,000 population in 1999 to 2.0 in 2009, with an APC of 0.19 (95% CI: −0.33 to 0.71), indicating a relatively stable trend with no significant change. However, after 2009, the AAMR showed a gradual but more pronounced increase, reaching 2.40 in 2020, with a statistically significant APC of 1.26 (CI: 0.80 to 1.71; see Table S2, which demonstrates the APC increase). This suggests that while NIP-related mortality remained relatively stable in the early years, there was a more consistent upward trend in later years.

### 4.1. NIP – related AAMR stratified via sex

**Figure 2.**
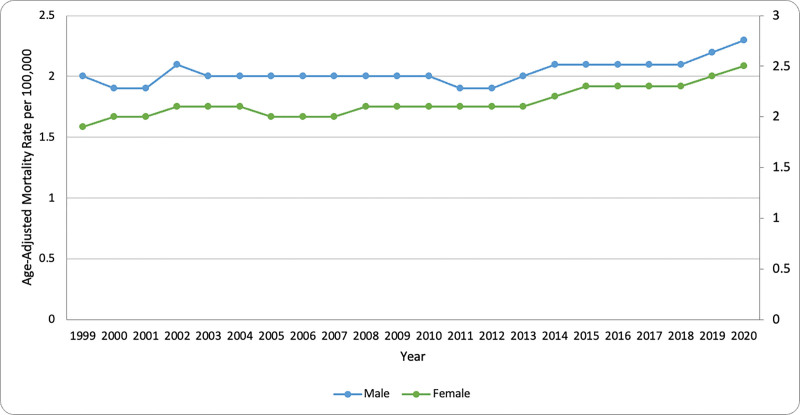
Trends in NIP-related mortality by gender in the U.S. (1999–2020). Line graph showing age-adjusted mortality rates (AAMR) per 100,000 amongst males and females. AAMR = age-adjusted mortality rate, NIP = nontraumatic intestinal perforation.

The AAMR from 1999 to 2020 exhibits a persistent sex-based disparity, with males consistently having higher mortality rates than females (Fig. 2; see Table S1, which demonstrates higher mortality in males). While the results generated for both groups show a general upward incline in trend over the study period, the projected increase was more pronounced amongst males. The male mortality rate fluctuated around 4.0 per 100,000 in the early 2000s, with a slight decrease in the late 2000s and soon followed via a gradual rise from 2012 onwards, reaching approximately 5.0 per 100,000 by 2020. In comparison, female mortality rates remained relatively stable around 2.0 per 100,000, with minor fluctuations before showing a gradual increase after 2014. The overall trend suggests a widening gap between male and female mortality rates over time, particularly in the most recent years of observation. Overall, the AAMR was lower in females compared to males in 1999 (2.0 vs 1.90; CI: 1.90 to 2.0; *P* < .05) through to 2020, though across both sexes, females showed more of a consistent increase in AAMR over time (Table 1; see Table S3, which demonstrates lower AAMR in females).

**Table 1:** Annual percent change (APC) stratified by sex from 1999 to 2020.

Cohort	Segment	Jointpoint	APC (%)	Lower CI	Upper CI	*P*-value
Female	1	1	0.45*	0.05	0.86	.030
Female	2	1	1.93*	1.09	2.78	.000
Male	1	1	−0.07*	−0.50	0.35	.718
Male	2	1	1.63*	0.73	2.53	.001

APC = annual percent change, CI = confidence interval.

* Indicates that the annual percent change is significantly different from zero at the α = 0.05 level.

Data source: Data derived from the CDC WONDER database, Centers for Disease Control and Prevention.^[5]^

### 4.2. NIP – related AAMR stratified via race

**Figure 3.**
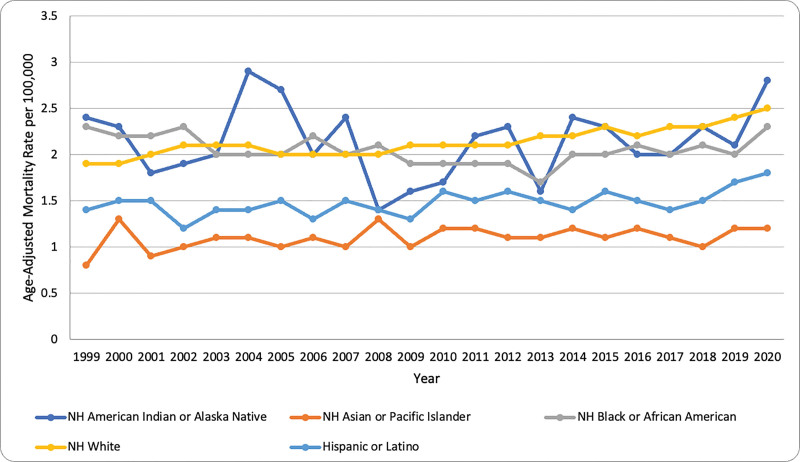
Trends in NIP-related mortality in different ethnic groups in the U.S. (1999–2020). Line graph showing age-adjusted mortality rates (AAMR) per 100,000 in different ethnic groups. AAMR = age-adjusted mortality rate, NH = non-Hispanic, NIP = nontraumatic intestinal perforation.

The APC values highlighted in Table 2 (see Table S2, which demonstrates the APC across racial groups) notable patterns among racial groups: the White cohort peaked at 3.15% (*P* < .05), the Black or African American group saw an early drop of −1.42% followed by a rebound to 2.60% (*P* > .05), and the Hispanic or Latino group showed a consistent steady increase of 0.80% (*P* < .05). AAMRs were higher in patients reported as NH compared to Hispanic/Latinos (2.10 vs 1.50; CI: 2.10 to 2.10; *P* < .05; Fig. 3). Throughout the study period, Hispanic or Latino populations exhibited the highest mortality rates, fluctuating between 7.9 and 11 per 100,000. This was further followed by NH White individuals demonstrating intermediate mortality rates, generally ranging between 6 and 8 per 100,000. The AAMR for NH Black or African American individuals, remained around 6 per 100,000 with varied fluctuations. Additionally, the NH Indian or Alaska Native populations and NH Asian or Pacific Islander populations consistently exhibited the lowest mortality rates, with values typically below 4 per 100,000. A similar pattern shown in Figure 3, are frequent fluctuations with AAMR values consistently rising and falling in a cyclical pattern every 2 to 3 years, across all racial groups. Moreover, a notable trend across all racial groups is an overall increase in mortality rates after 2019, with a particularly sharp increase in 2020, suggesting a significant external factor influencing mortality in the final years of observation (see Table S3, which demonstrates AAMR across races).

**Table 2:** Annual percent change (APC) stratified by race from 1999 to 2020.

Cohort	Segment	APC (%)	Lower CI	Upper CI	*P*-value
American Indian or Alaska Native	1	0.14	−1.20	1.50	.831
Asian or Pacific Islander	1	0.76*	−0.01	1.53	.052
Black or African American	1	−1.42*	−2.02	−0.83	.754
Black or African American	2	2.60	0.84	4.39	
White	1	3.15*	1.21	5.14	.007
White	2	−2.56	−8.26	3.49	
White	3	1.48*	1.19	1.76	
Hispanic or Latino	1	0.80*	0.28	1.33	.005

APC = annual percent change, CI = confidence interval.

* Indicates that the annual percent change is significantly different from zero at α = 0.05 level.

Data source: Data derived from the CDC WONDER database, Centers for Disease Control and Prevention.^[5]^

### 4.3. Annual trends for NIP-related AAMR via urbanization

**Figure 4.**
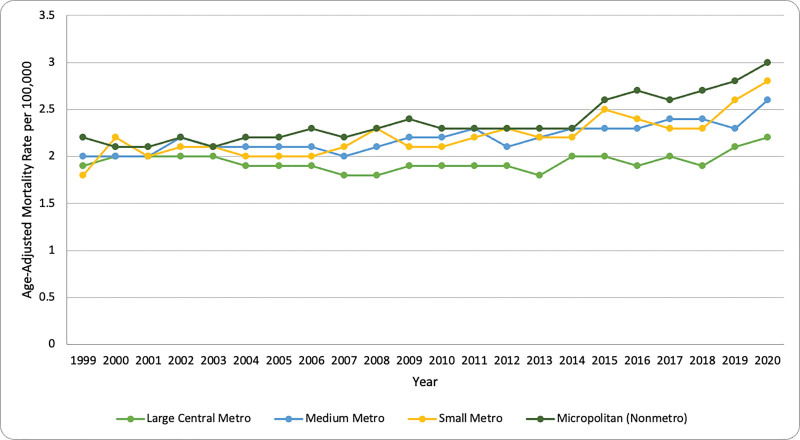
Trends in NIP-related mortality by urbanization level in the U.S. (1999–2020). Line graph showing age-adjusted mortality rates (AAMR) per 100,000 in micropolitan, metropolitan, and rural areas. AAMR = age-adjusted mortality rate, NIP = nontraumatic intestinal perforation.

From 1999 to 2020, there were clear disparities for the AAMR for various urbanized categories, with the highest rates being documented in micropolitan (nonmetro; 95% CI: 2.8–3.2; *P* < .05) in 2020, indicating a widening disparity in mortality rates based on urbanization levels (Fig. 4). The lowest mortality rates were seen in the large central metro areas. Additionally, in large central metro areas, the AAMR remained relatively stable between 1.8 and 2.2 per 100,000, with a peak of 2.2 (95% CI: 2.1–2.3; *P* < .05) in 2020 (Table 3). Positive values for the APC were observed in almost all depicted regions excluding the large central metropolitan area (APC: −0.43), which exhibited a slight decline overall. Over the period stated, mortality rates in the micropolitan areas exhibited a steady increase, with an evident rise after 2015 and a sharp peak in incline, within the year 2020. A similar pattern of distribution was observed in small and medium metro areas, where mortality rates began to increase following 2013. AAMR amongst Medium metro areas remained relatively constant, with only minor fluctuations exhibited up until 2020 (*P* < .05). Large central metro areas consistently had the lowest mortality rates. Overall, all areas reported an exponential growth in mortality rates within the year 2020 (see Table S4, which demonstrates the AAMR across urbanized/nonurban areas).

**Table 3:** Annual percent change (APC) stratified via urbanization from 1999 to 2020.

Cohort	Segment	Jointpoint	APC (%)	Lower CI	Upper CI	*P*-value
Large Central Metro	1	1	−0.43	−0.93	0.08	.090
Large Central Metro	2		1.97*	0.61	3.35	.007
Medium Metro	1	1	0.65*	0.12	1.18	.018
Medium Metro	2		1.7*	0.38	3.05	.015
Small Metro	1	1	0.97*	0.52	1.43	.000
Small Metro	2		8.79	−3.74	22.96	.165
Micropolitan (nonmetro)	1	1	0.69*	0.23	1.17	.006
Micropolitan (nonmetro)	2		3.31*	2.16	4.47	.000

APC = annual percent change, CI = confidence interval.

* Indicates that the annual percent change is significantly different from zero at α = 0.05 level.

Data source: Data derived from the CDC WONDER database, Centers for Disease Control and Prevention.^[5]^

### 4.4. NIP – related AAMR stratified via geographical region

**Figure 5.**
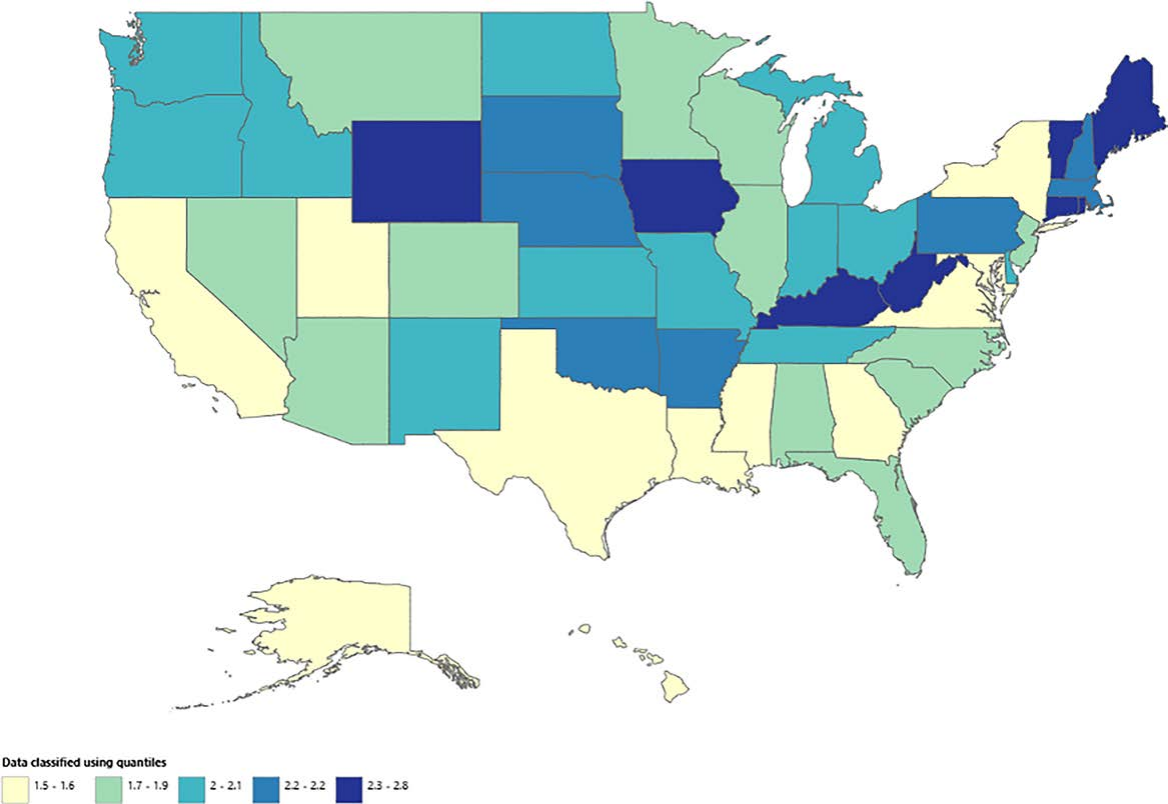
NIP-related mortality varied by census region in the U.S. (1999–2020). Map showing the age-adjusted mortality rates (AAMR) per 100,000 across the U.S. AAMR = age-adjusted mortality rate, NIP = nontraumatic intestinal perforation.

The AAMR for NIP varied by census region from 1999 to 2020 as illustrated in Figure 5. Values ranged from as low as 1.4 per 1000,000 in Florida (95% CI: 1.4–1.6), Kansas (95% CI: 1–2) and Mississippi (95% CI: 0.8–1.6), to a high of 4.1 in West Virginia (95% CI: 3.1–5.2). States which lie within the top 90th percentile included West Virginia (4.1), Iowa (4), New Mexico (3.7), Indiana (3.4), and Nebraska (3.4), rates exceeding nearly 3 times greater than states in the 10th percentile; Mississippi (1.1), Florida (1.3), and Kansas (1.4), highlighting a substantial variation within the data (see Tables S5 and S6, which demonstrates the AAMR and mortality across census regions). The spatial distribution suggests potential regional disparities influenced by demographic, socioeconomic, or healthcare access factors, warranting further investigation into underlying causes.

## 5. Discussion

**Figure 6.**
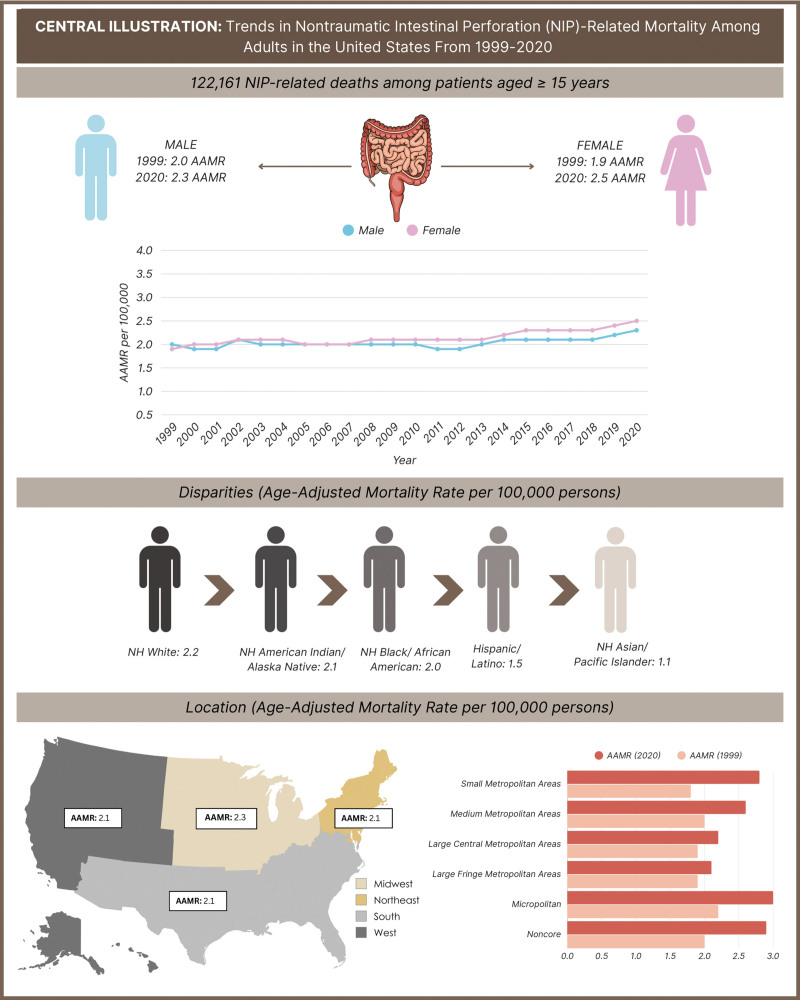
Central illustration – trends in NIP-related mortality among adults in the United States from 1999 to 2020. AAMR = age-adjusted mortality rate, NH = non-Hispanic, NIP = nontraumatic intestinal perforation.

Our study demonstrates significant epidemiological variations in the adult population. The overall AAMR has increased consistently over our study period (1999–2020), with a more pronounced increase observed after 2009 (Fig. 6). This pattern, in part, may be explained by the broader utilization of colonoscopy procedures in adults for conditions such as inflammatory bowel disease and polypectomy.^[11]^ However, whilst colonoscopy procedures in adults have increased substantially over recent decades, this factor alone is unlikely to explain the higher than normal number of reported NIP-related deaths. Increased procedural rates have likely contributed to better detection and reporting of NIP, yet large population-based studies indicate that the absolute risk of perforation and mortality following colonoscopy has remained low and stable over time. Therefore, the observed rise in NIP-related deaths likely reflects factors other than the procedural frequency, such as comorbidities, disease severity or changes in reporting practice. From a public health perspective, these findings highlight the need to more closely monitor patients at high risk, ensure more thorough pre- and post-procedural evaluation and strengthen hospital systems to prevent complications.^[12–14]^

When stratified by sex, males consistently exhibited higher AAMR than females, with the upward trend being more evident in the male population. This disparity may be attributed to lifestyle factors such as tobacco and alcohol consumption, which are historically higher among men. In 2022, 13.1% of U.S. men smoked cigarettes compared to 10.1% of women,^[15]^ and in 2023, 24.3% of men reported past-month binge drinking compared to 19.2% of women.^[16]^ These behaviors contribute to a higher incidence of peptic ulcer disease in men, which can progress to perforation and result in increased mortality.^[17–19]^

Minor fluctuations were observed in earlier years, particularly among males in the early 2000s, followed by a gradual rise from 2012 onward and peaking in 2020. A similar upward trend was seen among females after 2014, also reaching a peak in 2020. The persistent gap between male and female AAMR, especially in recent years, may reflect delayed healthcare-seeking behavior and more severe disease presentation among men, both of which are associated with higher mortality.^[20,21]^

Race-stratified trends indicated that the Hispanic or Latino population has the highest AAMRs among all races from 1999 to 2020. Hispanic patients with ulcerative colitis exhibited higher rates (2.51%) of severe rectal bleeding compared to other racial groups (1.79% in white population). This mucosal injury predisposed them to intestinal perforation.^[22]^ The lowest AAMR due to NIP was observed in NH American Indian or Alaska Native. A common point between all races was that they showed a similar fluctuation pattern every 2 to 3 years. There was an abrupt increase in mortality from 2019, with its peak achieved in 2020.

In terms of urbanization, disparities in our study were also evident. The highest mortality rates were recorded in micropolitan (nonmetro) areas and the lowest in large central metro areas. Furthermore, the trend has shown greater stability over the entire study period for large central metro areas. These differences may stem from delays in diagnosis and limited treatment of GI emergencies in rural areas, which is attributed to limited access to advanced imaging, a shortage of surgical teams, as well as longer transport hours to medical care. Such barriers are not prevalent in urban areas.^[23,24]^ The micropolitan areas had also experienced a steady rise until 2015, but in the subsequent year, there was a sharp rise, and the peak was reached in 2020. A similar trend was observed in small metro areas, where the sudden increase was observed following 2013. In medium metro areas, the trend has been relatively constant with some minor fluctuations. A consistent pattern across all areas was the peak observed in 2020, which could be associated with COVID-19. Studies have reported the incidence of large intestinal perforation in COVID-19 patients; this may be associated with increased inflammatory reactions, coagulopathy, medication effects (corticosteroids and immunomodulators), or direct viral injury.^[25]^

Regarding geographic disparities, differences were not highly significant; nonmetropolitan areas had higher AAMR compared to metropolitan areas likely due to limited access to healthcare services, including intensive care units and emergency surgical care necessary to prevent mortality.^[26]^ The highest mortality rates were concentrated in the Midwest, Northeast, and parts of the South potentially due to the highest prevalence of risk factors, including smoking, alcohol use, and comorbidities (e.g., diabetes and cardiovascular disease). Additionally, socioeconomic factors and variations in healthcare infrastructure variability also contribute the differences.^[26]^ The lowest mortality rates were recorded in the Western and Southern regions which may be linked to higher rates of insurance coverage, better healthcare infrastructure, and more widespread preventive care strategies in place.^[27]^

Our study has certain limitations. First, the data extraction was solely based on International Statistical Classification of Diseases and Related Health Problems-10th Revision codes assigned by WHO which may lead to misclassification or omissions; in addition, reliance on death certificate data introduces possible inaccuracies in identifying the true underlying cause of death. Second, the data’s validity may be affected by inconsistent documentation practices across the healthcare institutions in the U.S. Third, the dataset lacked individual patient-specific clinical information such as treatment history, and certain confounding factors such as socioeconomic status and access to healthcare. Lastly, there may be potential reporting biases and differences in healthcare quality across regions. Despite these limitations, the study’s strength is in using a large, nationally representative dataset from the CDC WONDER Analysis and a long observation period, offering valuable context to understand the changing epidemiology of NIP.

## 6. Conclusion

Our nationwide analysis shows a steady rise in NIP-related mortality from 1999 to 2020, which sharp increases after 2009 and in 2020. AAMR was highest among males, Hispanic individuals, and residents of nonmetropolitan areas. States like West Virginia and Iowa reported rates nearly triple those in Florida or Mississippi. Limited surgical access, lifestyle-related risks, and delayed diagnosis, among various other factors, likely drive these disparities. Our findings call for region-specific healthcare planning, earlier detection strategies, and improved emergency surgical access in underserved communities.

## Author contributions

**Conceptualization:** Onyekachi Emmanuel Anyagwa, Hashim Mohamed Siraj, Muskan Joshi, Mohammad Aamir Qayyum Sarguroh Khan, Muhammad Husnain Ahmad, Nivedita Pant, Hanzala Zahid, Areeba Zubair.

**Data curation:** Oluwatoyin Adalia Dairo, Mohammad Aamir Qayyum Sarguroh Khan.

**Methodology:** Abhirami Babu.

**Validation:** Mohammad Aamir Qayyum Sarguroh Khan, Masab Ali, Nivedita Pant, Hanzala Zahid, Areeba Zubair, Chinedu Elvis Nwaduru.

**Visualization:** Oluwatoyin Adalia Dairo, Abhirami Babu, Nivedita Pant, Hanzala Zahid, Chinedu Elvis Nwaduru.

**Writing – original draft:** Onyekachi Emmanuel Anyagwa, Hashim Mohamed Siraj, Muskan Joshi, Oluwatoyin Adalia Dairo, Mohammad Aamir Qayyum Sarguroh Khan, Anas Abdulkader, Muhammad Husnain Ahmad, Masab Ali, Abhirami Babu, Areeba Zubair, Chinedu Elvis Nwaduru.

**Writing – review & editing:** Muskan Joshi, Anas Abdulkader, Muhammad Husnain Ahmad, Masab Ali, Hanzala Zahid.












