# Supplementary material for: Trends in nontraumatic intestinal perforation-related mortality among adults in the United States from 1999 to 2020: A nationwide CDC WONDER analysis
Source: Medicine (Baltimore). 2026 May 22;105(21):e48931. doi: 10.1097/MD.0000000000048931 (PMC13200925; doi:10.1097/MD.0000000000048931)
Supplement: Supplementary file 1 [file medi-105-e48931-s001.docx]

**Supplemental Digital Content, Table 2:** Nontraumatic Intestinal Perforation Mortality-Related Annual Percentage Change per 100,000 in Adults in the United States, 1999 to 2020

| **Annual Percentage Change (APC)** | |
| --- | --- |
| **Year Interval** | **APC (95% CI)** |
| **Men** | |
| 1999-2012 | -0.07 (-0.49 to 0.35) |
| 2012-2020 | 1.62* (0.73 to 2.53) |
| **Women** | |
| 1999-2006 | 0.23 (-8.05 to 8.97) |
| 2006 – 2020 | 1.44* (1.13 to 1.75) |
| **NH White** | |
| 1999-2012 | 0.58* (0.12 to 1.04) |
| 2012-2020 | 1.81* (0.84 to 2.79) |
| **NH Black or African American** | |
| 1999 - 2013 | -1.42* (-2.02 to -0.82) |
| 2013-2020 | 2.59* (0.83 to 4.38) |
| **NH American Indian or Alaska Native** | |
| 1999-2009 | -2.41 (-6.51 to 1.85) |
| 2009-2020 | 2.42 (-1.3 to 6.3) |
| **Hispanic or Latino** | |
| 1999-2018 | 0.42 (-0.2 to 1.04) |
| 2018-2020 | 9.38 (-8.08 to 30.16) |
| **NH Asian or Pacific Islander** | |
| 1999-2008 | 1.81 (-1.09 to 4.86) |
| 2008-2020 | 0.05 (-1.82 to 1.96) |
| **Urban** | |
| 1999-2002 | 3.22* (0.11 to 6.43) |
| 2002-2007 | -1.56 (-3.36 to 0.27) |
| 2007-2020 | 1.42* (1.13 to 1.72) |
| **Rural** | |
| 1999-2014 | 0.90* (0.53 to 1.27) |
| 2014-2020 | 3.40* (2.19 to 4.62) |

*Indicates that the APC is significantly different from zero at the alpha = 0.05 level.

**NH: Non Hispanics**

APC: **Annual Percentage Change**
