# Supplementary material for: Trends in nontraumatic intestinal perforation-related mortality among adults in the United States from 1999 to 2020: A nationwide CDC WONDER analysis
Source: Medicine (Baltimore). 2026 May 22;105(21):e48931. doi: 10.1097/MD.0000000000048931 (PMC13200925; doi:10.1097/MD.0000000000048931)
Supplement: Supplementary file 2 [file medi-105-e48931-s002.docx]

**Supplemental Digital Content, Table 1:** Nontraumatic Intestinal Perforation-Related Mortality, Stratified by Sex and Race, in Adults in the United States, 1999 to 2020

| **Mortality** | | | | | | | | |
| --- | --- | --- | --- | --- | --- | --- | --- | --- |
| **Year** | **Overall** | **Women** | **Men** | **NH White** | **NH Black or African American** | **NH Asian or Pacific Islander** | **NH American Indian or Alaska Native** | **Hispanic or Latino** |
| 1999 | 4181 | 2482 | 1699 | 3677 | 434 | 44 | 26 | 168 |
| 2000 | 4268 | 2616 | 1652 | 3758 | 416 | 67 | 27 | 176 |
| 2001 | 4322 | 2635 | 1687 | 3833 | 414 | 52 | 23 | 190 |
| 2002 | 4657 | 2791 | 1866 | 4117 | 457 | 58 | 25 | 164 |
| 2003 | 4666 | 2807 | 1859 | 4163 | 413 | 68 | 22 | 189 |
| 2004 | 4702 | 2808 | 1894 | 4164 | 426 | 75 | 37 | 200 |
| 2005 | 4754 | 2838 | 1916 | 4201 | 438 | 75 | 40 | 221 |
| 2006 | 4822 | 2850 | 1972 | 4232 | 476 | 85 | 29 | 211 |
| 2007 | 4837 | 2818 | 2019 | 4263 | 451 | 83 | 40 | 235 |
| 2008 | 5114 | 3019 | 2095 | 4497 | 482 | 109 | 26 | 247 |
| 2009 | 5189 | 3089 | 2100 | 4615 | 454 | 93 | 27 | 247 |
| 2010 | 5317 | 3111 | 2206 | 4705 | 464 | 117 | 31 | 311 |
| 2011 | 5506 | 3289 | 2217 | 4860 | 477 | 125 | 44 | 311 |
| 2012 | 5603 | 3318 | 2285 | 4938 | 500 | 120 | 45 | 344 |
| 2013 | 5827 | 3414 | 2413 | 5183 | 485 | 127 | 32 | 347 |
| 2014 | 6124 | 3563 | 2561 | 5365 | 557 | 147 | 55 | 336 |
| 2015 | 6501 | 3817 | 2684 | 5705 | 600 | 143 | 53 | 414 |
| 2016 | 6590 | 3850 | 2740 | 5731 | 631 | 173 | 55 | 412 |
| 2017 | 6839 | 4035 | 2804 | 6010 | 614 | 161 | 54 | 415 |
| 2018 | 7033 | 4118 | 2915 | 6147 | 665 | 161 | 60 | 464 |
| 2019 | 7446 | 4346 | 3100 | 6523 | 676 | 190 | 57 | 526 |
| 2020 | 7863 | 4579 | 3284 | 6776 | 794 | 205 | 88 | 598 |
| **Total** | **122161** | **72193** | **49968** | **107463** | **11324** | **2478** | **896** | **6727** |

NH: Non hispanic
