# Supplementary material for: Trends in nontraumatic intestinal perforation-related mortality among adults in the United States from 1999 to 2020: A nationwide CDC WONDER analysis
Source: Medicine (Baltimore). 2026 May 22;105(21):e48931. doi: 10.1097/MD.0000000000048931 (PMC13200925; doi:10.1097/MD.0000000000048931)
Supplement: Supplementary file 3 [file medi-105-e48931-s003.docx]

**Supplemental Digital Content, Table 3:** Nontraumatic Intestinal Perforation-Related Age-Adjusted Mortality Rates per 100,000 Stratified by Gender and Race in Adults in the United States, 1999 to 2020

| **Age Adjusted Mortality Rate (95% CI)** | | | | | | | |
| --- | --- | --- | --- | --- | --- | --- | --- |
| **Year** | **Women** | **Men** | **NH White** | **NH Black or African American** | **NH Asian or Pacific Islander** | **NH American Indian or Alaska Native** | **Hispanic or Latino** |
| 1999 | 1.9 (1.9-2.0) | 2 (1.9 - 2.1) | 1.9 (1.9 – 2.0) | 2.3 (2.1 – 2.5) | 0.8 (0.6 – 1.1) | 2.4 (1.5 – 3.6) | 1.4 (1.2 – 1.7) |
| 2000 | 2.0 (2.0 – 2.1) | 1.9 (1.8 - 2.0) | 1.9 (1.9 – 2.0) | 2.2 (1.9 – 2.4) | 1.3 (1 – 1.7) | 2.3 (1.5 – 3.5) | 1.5 (1.3 – 1.8) |
| 2001 | 2.0 (1.9-2.1) | 1.9 (1.8 - 2.0) | 2 (1.9 – 2.0) | 2.2 (2 – 2.4) | 0.9 (0.7 – 1.2) | 1.8 (1.1 – 2.8) | 1.5 (1.3 – 1.8) |
| 2002 | 2.1 (2-2.2) | 2.1 (2.0 - 2.2) | 2.1 (2.0 – 2.2) | 2.3 (2.1 – 2.5) | 1 (0.7 – 1.3) | 1.9 (1.2 – 2.9) | 1.2 (1.0 – 1.4) |
| 2003 | 2.1 (2.0 - 2.2) | 2.0 (1.9 - 2.1) | 2.1 (2.0 – 2.2) | 2.0 (1.8 – 2.2) | 1.1 (0.8 – 1.4) | 2 (1.2 – 3.1) | 1.4 (1.1 – 1.6) |
| 2004 | 2.1 (2.0 - 2.1) | 2.0 (1.9 - 2.1) | 2.1 (2.0 – 2.2) | 2.0 (1.8 – 2.2) | 1.1(0.9 - 1.4) | 2.9 (2 – 4.1) | 1.4 (1.2 – 1.6) |
| 2005 | 2.0 (2.0 – 2.1) | 2.0 (1.9 - 2.1) | 2.0 (2.0 – 2.1) | 2 (1.8 – 2.2) | 1 (0.8 – 1.2) | 2.7 (1.8 – 3.7) | 1.5 (1.3 – 1.7) |
| 2006 | 2.0 (1.9 – 2.1) | 2.0 (1.9 - 2.1) | 2.0 (2.0 – 2.1) | 2.2 (2 – 2.4) | 1.1 (0.9 - 1.4) | 2 (1.3 – 2.9) | 1.3 (1.1 – 1.5) |
| 2007 | 2.0 (1.9 – 2.0) | 2.0 (1.9 - 2.1) | 2.0 (1.9 – 2.1) | 2 (1.8 – 2.2) | 1 (0.8 – 1.2) | 2.4 (1.7 – 3.4) | 1.5 (1.3 – 1.6) |
| 2008 | 2.1 (2.0 - 2.2) | 2.0 (1.9 - 2.1) | 2.0 (2.0 – 2.1) | 2.1 (1.9 – 2.3) | 1.3 (1 – 1.5) | 1.4 (0.9 – 2.1) | 1.4 (1.2 – 1.6) |
| 2009 | 2.1 (2.0 - 2.2) | 2.0 (1.9 - 2.0) | 2.1 (2.0 – 2.2) | 1.9 (1.7 – 2.1) | 1 (0.8 – 1.3) | 1.6 (1 – 2.4) | 1.3 (1.1 – 1.5) |
| 2010 | 2.1 (2.0 – 2.1) | 2.0 (1.9 - 2.1) | 2.1 (2.0 – 2.2) | 1.9 (1.7 – 2.1) | 1.2 (1 – 1.4) | 1.7 (1.1 – 2.4) | 1.6 (1.4 – 1.8) |
| 2011 | 2.1 (2.1 – 2.2) | 1.9 (1.9 – 2.0) | 2.1 (2.1 – 2.2) | 1.9 (1.7 – 2.1) | 1.2 (1 – 1.4) | 2.2 (1.5 – 3) | 1.5 (1.4 – 1.7) |
| 2012 | 2.1 (2.0 – 2.2) | 1.9 (1.9 – 2.0) | 2.1 (2 .0 – 2.2) | 1.9 (1.7 – 2.1) | 1.1 (0.9 – 1.3) | 2.3 (1.7 – 3.2) | 1.6 (1.4 – 1.8) |
| 2013 | 2.1 (2.1 – 2.2) | 2.0 (1.9 - 2.1) | 2.2 (2.1 – 2.3) | 1.7 (1.6 – 1.9) | 1.1 (0.9 – 1.3) | 1.6 (1.1 – 2.3) | 1.5 (1.4 – 1.7) |
| 2014 | 2.2 (2.1 – 2.2) | 2.1 (2.0 - 2.1) | 2.2 (2.1 – 2.2) | 2 (1.8 – 2.2) | 1.2 (1 – 1.4) | 2.4 (1.8 – 3.1) | 1.4 (1.2 – 1.6) |
| 2015 | 2.3 (2.2 – 2.4) | 2.1 (2.0 - 2.2) | 2.3 (2.2 – 2.4) | 2 (1.9 – 2.2) | 1.1 (0.9 – 1.3) | 2.3 (1.7 – 3) | 1.6 (1.5 – 1.8) |
| 2016 | 2.3 (2.2 - 2.3) | 2.1 (2.0 - 2.2) | 2.2 (2.2 – 2.3) | 2.1 (1.9 – 2.2) | 1.2 (1 – 1.4) | 2.0 (1.5 – 2.7) | 1.5 (1.3 – 1.6) |
| 2017 | 2.3 (2.2 - 2.4) | 2.1 (2.0 - 2.2) | 2.3 (2.2 – 2.4) | 2.0 (1.8 – 2.1) | 1.1 (0.9 – 1.3) | 2.0 (1.5 – 2.7) | 1.4 (1.3 – 1.5) |
| 2018 | 2.3 (2.2 - 2.4) | 2.1 (2.0 - 2.2) | 2.3 (2.3 – 2.4) | 2.1 (1.9 – 2.2) | 1 (0.9 – 1.2) | 2.3 (1.7 – 2.9) | 1.5 (1.4 – 1.7) |
| 2019 | 2.4 (2.3 - 2.5) | 2.2 (2.1 - 2.2) | 2.4 (2.4 – 2.5) | 2.0 (1.8 – 2.1) | 1.2 (1 – 1.3) | 2.1 (1.5 – 2.7) | 1.7 (1.5 – 1.8) |
| 2020 | 2.5 (2.4 - 2.6) | 2.3 (2.2 - 2.4) | 2.5 (2.4 – 2.6) | 2.3 (2.1 – 2.5) | 1.2 (1 – 1.3) | 2.8 (2.2 – 3.5) | 1.8 (1.6 – 1.9) |
| **Total** | **2.2 (2.1 – 2.2)** | **2.0 (2.0 – 2.1)** | **2.2 (2.1 – 2.2)** | **2.0 (2.0 – 2.1)** | **1.1 (1.1 - 1.2)** | **2.1 (2.0 – 2.3)** | **1.5 (1.5 – 1.5)** |

NH = non-Hispanic.
