# Supplementary material for: Trends in nontraumatic intestinal perforation-related mortality among adults in the United States from 1999 to 2020: A nationwide CDC WONDER analysis
Source: Medicine (Baltimore). 2026 May 22;105(21):e48931. doi: 10.1097/MD.0000000000048931 (PMC13200925; doi:10.1097/MD.0000000000048931)
Supplement: Supplementary file 4 [file medi-105-e48931-s004.docx]

**Supplemental Digital Content, Table 4:** Nontraumatic Intestinal Perforation-Related Age-Adjusted Mortality Rates per 100,000 Stratified by Urban-Rural Classification in Adults in the United States, 1999 to 2020

| **Year** | **Urban** | **Rural** |
| --- | --- | --- |
| 1999 | 1.9 (1.8 - 2.0) | 2.1 (1.9 – 2.3) |
| 2000 | 2.0 (1.9 - 2.1) | 2.0 (1.8 – 2.2) |
| 2001 | 2.0 (1.8 - 2.1) | 2.1 (1.9 – 2.3) |
| 2002 | 2.1 (1.9 - 2.2) | 2.3 (2.0 – 2.5) |
| 2003 | 2.1 (1.9 - 2.2) | 2.2 (2.0 – 2.4) |
| 2004 | 2.0 (1.9 - 2.2) | 2.2 (2.0 – 2.4) |
| 2005 | 2.0 (1.9 - 2.1) | 2.2 (2.0 – 2.4) |
| 2006 | 2.0 (1.9 - 2.1) | 2.3 (2.1 – 2.5) |
| 2007 | 1.9 (1.8 - 2.1) | 2.2 (2.0 – 2.4) |
| 2008 | 2.0 (1.9 - 2.2) | 2.3 (2.1 – 2.6) |
| 2009 | 2.0 (1.9 - 2.1) | 2.3 (2.1 – 2.5) |
| 2010 | 2.0 (1.9 - 2.2) | 2.3 (2.1 – 2.5) |
| 2011 | 2.1 (1.9 - 2.2) | 2.3 (2.1 – 2.5) |
| 2012 | 2.1 (1.9 - 2.2) | 2.4 (2.2 – 2.6) |
| 2013 | 2.1 (1.9 - 2.2) | 2.4 (2.2 – 2.6) |
| 2014 | 2.1 (2.0 - 2.3) | 2.3 (2.1 – 2.6) |
| 2015 | 2.2 (2.1 - 2.3) | 2.6 (2.4 – 2.8) |
| 2016 | 2.2 (2.1 - 2.3) | 2.6 (2.4 – 2.8) |
| 2017 | 2.2 (2.1 - 2.3) | 2.6 (2.4 – 2.8) |
| 2018 | 2.2 (2.1 - 2.3) | 2.7 (2.5 – 2.9) |
| 2019 | 2.3 (2.2 - 2.4) | 2.8 (2.6 – 3.0) |
| 2020 | 2.4 (2.3 - 2.6) | 3.0 (2.8 – 3.2) |
| **Total** | 2.1 (1.9 – 2.2) | 2.4 (2.2 – 2.6) |
