# Supplementary material for: Trends in nontraumatic intestinal perforation-related mortality among adults in the United States from 1999 to 2020: A nationwide CDC WONDER analysis
Source: Medicine (Baltimore). 2026 May 22;105(21):e48931. doi: 10.1097/MD.0000000000048931 (PMC13200925; doi:10.1097/MD.0000000000048931)
Supplement: Supplementary file 5 [file medi-105-e48931-s005.docx]

**Supplemental Digital Content, Table 5:** Nontraumatic Intestinal Perforation-Related Age-Adjusted Mortality Rates per 100,000 Stratified by Census Region in Adults in the United States, 1999 to 2020

| **Census Region** | **Year** | **Age-Adjusted Rate (95% CI)** |
| --- | --- | --- |
| **Northeast** | 1999 | 1.9 (1.8 – 2.0) |
| **Northeast** | 2000 | 1.9 (1.8 – 2.1) |
| **Northeast** | 2001 | 1.8 (1.7 – 2) |
| **Northeast** | 2002 | 2.1 (2.0 – 2.3) |
| **Northeast** | 2003 | 2.1 (2.0 – 2.3) |
| **Northeast** | 2004 | 2 (1.9 – 2.2) |
| **Northeast** | 2005 | 2.1 (2.0 – 2.2) |
| **Northeast** | 2006 | 2.1 (1.9 – 2.2) |
| **Northeast** | 2007 | 1.9 (1.8 – 2.0) |
| **Northeast** | 2008 | 1.9 (1.8 – 2.0) |
| **Northeast** | 2009 | 2.0 (1.9 – 2.1) |
| **Northeast** | 2010 | 2.0 (1.9 – 2.1) |
| **Northeast** | 2011 | 2.0 (1.9 – 2.1) |
| **Northeast** | 2012 | 2.1 (2.0 – 2.2) |
| **Northeast** | 2013 | 2.1 (2.0 – 2.2) |
| **Northeast** | 2014 | 2.2 (2.1 – 2.3) |
| **Northeast** | 2015 | 2.2 (2.1 – 2.3) |
| **Northeast** | 2016 | 2.1 (1.9 – 2.2) |
| **Northeast** | 2017 | 2.2 (2.1 – 2.3) |
| **Northeast** | 2018 | 2.2 (2.1 – 2.3) |
| **Northeast** | 2019 | 2.3 (2.1 – 2.4) |
| **Northeast** | 2020 | 2.3 (2.1 – 2.4) |
| **Total** | | 2.1 (2.0 – 2.1) |
| **Midwest** | 1999 | 2.0 (1.9 – 2.1) |
| **Midwest** | 2000 | 2.1 (2.0 – 2.2) |
| **Midwest** | 2001 | 2.1 (2.0 – 2.2) |
| **Midwest** | 2002 | 2.3 (2.1 – 2.4) |
| **Midwest** | 2003 | 2.2 (2.0 – 2.3) |
| **Midwest** | 2004 | 2.2 (2.0 – 2.3) |
| **Midwest** | 2005 | 2.2 (2.0 – 2.3) |
| **Midwest** | 2006 | 2.1 (2.0 – 2.2) |
| **Midwest** | 2007 | 2.1 (2.0 – 2.2) |
| **Midwest** | 2008 | 2.2 (2.1 – 2.3) |
| **Midwest** | 2009 | 2.1 (2.0 – 2.2) |
| **Midwest** | 2010 | 2.1 (2.0 – 2.2) |
| **Midwest** | 2011 | 2.2 (2.1 – 2.3) |
| **Midwest** | 2012 | 2.2 (2.1 – 2.3) |
| **Midwest** | 2013 | 2.3 (2.2 – 2.4) |
| **Midwest** | 2014 | 2.3 (2.2 – 2.4) |
| **Midwest** | 2015 | 2.3 (2.2 – 2.5) |
| **Midwest** | 2016 | 2.3 (2.2 – 2.4) |
| **Midwest** | 2017 | 2.5 (2.3 – 2.6) |
| **Midwest** | 2018 | 2.4 (2.2 – 2.5) |
| **Midwest** | 2019 | 2.4 (2.3 – 2.6) |
| **Midwest** | 2020 | 2.7 (2.5 – 2.8) |
| **Total** | | 2.3 (2.2 – 2.3) |
| **South** | 1999 | 2 (1.9 – 2.1) |
| **South** | 2000 | 1.9 (1.8 – 2.0) |
| **South** | 2001 | 2.0 (1.9 – 2.1) |
| **South** | 2002 | 2.0 (1.9 – 2.1) |
| **South** | 2003 | 1.9 (1.8 – 2.0) |
| **South** | 2004 | 2.0 (1.9 – 2.1) |
| **South** | 2005 | 1.9 (1.8 – 2.0) |
| **South** | 2006 | 2.0 (1.9 – 2.1) |
| **South** | 2007 | 2.0 (1.9 – 2.0) |
| **South** | 2008 | 2.0 (1.9 – 2.1) |
| **South** | 2009 | 2.0 (1.9 – 2.1) |
| **South** | 2010 | 2.0 (1.9 – 2.1) |
| **South** | 2011 | 2.0 (1.9 – 2.1) |
| **South** | 2012 | 2.0 (1.9 – 2.0) |
| **South** | 2013 | 2.0 (1.9 – 2.1) |
| **South** | 2014 | 2.1 (2.0 – 2.2) |
| **South** | 2015 | 2.2 (2.1 – 2.3) |
| **South** | 2016 | 2.2 (2.1 – 2.3) |
| **South** | 2017 | 2.2 (2.0 – 2.3) |
| **South** | 2018 | 2.2 (2.2 – 2.3) |
| **South** | 2019 | 2.3 (2.2 – 2.3) |
| **South** | 2020 | 2.3 (2.3 – 2.4) |
| **Total** | | 2.1 (2.0 – 2.1) |
| **West** | 1999 | 1.8 (1.7 – 1.9) |
| **West** | 2000 | 2.0 (1.8 – 2.1) |
| **West** | 2001 | 1.8 (1.7 – 2) |
| **West** | 2002 | 2.0 (1.9 – 2.1) |
| **West** | 2003 | 2.0 (1.9 – 2.1) |
| **West** | 2004 | 2.0 (1.9 – 2.1) |
| **West** | 2005 | 1.9 (1.8 – 2.0) |
| **West** | 2006 | 1.9 (1.8 – 2.0) |
| **West** | 2007 | 2.0 (1.8 – 2.1) |
| **West** | 2008 | 2.0 (1.9 – 2.1) |
| **West** | 2009 | 2.0 (1.9 – 2.1) |
| **West** | 2010 | 2.1 (2.0 – 2.2) |
| **West** | 2011 | 2.0 (1.9 – 2.2) |
| **West** | 2012 | 2.0 (1.9 – 2.1) |
| **West** | 2013 | 2.0 (1.9 – 2.1) |
| **West** | 2014 | 2.0 (1.9 – 2.1) |
| **West** | 2015 | 2.2 (2.1 – 2.3) |
| **West** | 2016 | 2.2 (2.1 – 2.3) |
| **West** | 2017 | 2.2 (2.1 – 2.3) |
| **West** | 2018 | 2.2 (2.1 – 2.3) |
| **West** | 2019 | 2.4 (2.2 – 2.5) |
| **West** | 2020 | 2.4 (2.3 – 2.5) |
| **Total** | | 2.1 (2.0 – 2.1) |
