# Supplementary material for: Trends in nontraumatic intestinal perforation-related mortality among adults in the United States from 1999 to 2020: A nationwide CDC WONDER analysis
Source: Medicine (Baltimore). 2026 May 22;105(21):e48931. doi: 10.1097/MD.0000000000048931 (PMC13200925; doi:10.1097/MD.0000000000048931)
Supplement: Supplementary file 6 [file medi-105-e48931-s006.docx]

**Supplemental Digital Content, Table 6:** Nontraumatic Intestinal Perforation-Related Mortality in Adults in the United States, stratified by States from 1999 to 2020

| **State** | **Death** |
| --- | --- |
| Alabama | 1968 |
| Alaska | 228 |
| Arizona | 2404 |
| Arkansas | 1350 |
| California | 11856 |
| Colorado | 1828 |
| Connecticut | 1814 |
| Delaware | 390 |
| District of Columbia | 198 |
| Florida | 7342 |
| Georgia | 3192 |
| Hawaii | 474 |
| Idaho | 686 |
| Illinois | 5044 |
| Indiana | 2918 |
| Iowa | 1624 |
| Kansas | 1243 |
| Kentucky | 2124 |
| Louisiana | 1535 |
| Maine | 713 |
| Maryland | 2029 |
| Massachusetts | 3205 |
| Michigan | 4480 |
| Minnesota | 2110 |
| Mississippi | 962 |
| Missouri | 2745 |
| Montana | 411 |
| Nebraska | 864 |
| Nevada | 1047 |
| New Hampshire | 617 |
| New Jersey | 3393 |
| New Mexico | 850 |
| New York | 6799 |
| North Carolina | 3844 |
| North Dakota | 306 |
| Ohio | 5229 |
| Oklahoma | 1754 |
| Oregon | 1780 |
| Pennsylvania | 6152 |
| Rhode Island | 616 |
| South Carolina | 1902 |
| South Dakota | 397 |
| Tennessee | 2896 |
| Texas | 8096 |
| Utah | 926 |
| Vermont | 338 |
| Virginia | 2729 |
| Washington | 3011 |
| West Virginia | 1106 |
| Wisconsin | 2356 |
| Wyoming | 280 |
| Total | 122161 |
